# 

**Published:** 1899-11-11

**Authors:** 


### NOTICE TO CORRESPONDENTS.

All MS., Letters, Books for Review, and other matters intended for the Editor should be addressed The Editor, "The Hospital," The Hospital Building, 28 & 29, Southampton Street, Strand, W.C.
The Editor cannot undertake to return rejected MS., even when accompanied by stamped directed envelope.

**Notice to Advertisers and Subscribers.**

All Advertisements, Orders for Copies of The Hospital, and Business Communications should be addressed to **THE MANAGER (Not to the Editor)**, The Hospital, The Hospital Building, 28 & 29, Southampton Street, Strand, London, W.C.
The Cost of Subscription, post free, is as follows:—Per week, 2½d.; per quarter, 2s. 8d.; per half-year, 5s. 3d.; per annum, 10s. 6d. Foreign:—Per annum, 15s. 2d., payable, in all cases, in advance.

**NOTICE.—Vol. XXVI. of "The Hospital" (April, 1899, to September, 1899), in handsome cloth binding, will shortly be on sale at the Office of this Journal, price 7s. 6d. Cases for binding the Numbers contained in this Volume 1s. 6d. Index to Vol. XXVI., 2½d., post free.**

**The Monthly Part for November is now ready. Price 6d., by post 8d.**

### BOOKS RECEIVED.

The Children's Book Room.

"Bubbles." Edited by Dr. Barnardo.

Henry J. Glaisher.

"Cancer: Illustrated by One Thousand Cases from the Register of the Middlesex Hospital." By Thomas William Nunn, F.R.C.S.

William Blackwood and Sons.

"Practical Nursing." By Isla Stewart and Herbert E. Cuff, M.D., F.R.C.S. In two volumes. Vol. I.

H. K. Lewis.

"A Contribution to the Surgery of Fractures and Dislocations of the Upper Extremity." By J. E. Platt, M.S.Lond., F.R.C.S.

Pewtriss and Co.

"Spencer's Disease: Dermatitis Multiformis Exfoliativa." By Walter Spencer, M.D.

Oliver and Boyd.

"A Manual of Modern Gastric Methods—Chemical, Physical, and Therapeutical." By A. Lockhart Gillespie, M.D., F.R.C.P.E. With a chapter on "The Mechanical Methods Used in Young Children." By John Thomson, M.D., F.R.C.P.Ed.

**Periodicals and Pamphlets.**—Medical Press—Sanitary Record—Guy's Hospital Gazette—Literary Digest—Municipal Journal—Hospital and Sanitarium Record—Vectis—Asylum News—Medical Record—Educational Review.

### The Student of Medicine.

### APPOINTMENTS.

A. Armer, M.B.Lond., appointed Assistant Medical Officer of the Workhouse for the Township of Toxteth Park. J. G. Boon, L.R.C.P., L.R.C.S.Irel., appointed Medical Officer for the Broseley District of the Madeley Union. A. P. Brown, L.R.C.P., L.R.C.S.Edin., L.F.P.S.Glasg., appointed Medical Officer for the Elsdon District of the Rothbury Union. J. J. Butterworth, M.B., B.Ch., appointed Junior House Surgeon to the Manchester Royal Eye Hospital. B. E. Church, L.S.A., appointed Medical Officer of the 4th District of the Stroud Union. J. G. Clegg, M.D., B.S., F.R.C.S., appointed Honorary Assistant Surgeon to the Manchester Royal Eye Hospital. S. Craddock, M.R.C.S.Eng., L.S.A., appointed Medical Officer to the Children's Homes of the Bath Union. J. Huskie, M.B., C.M.Edin., appointed Medical Officer of the North Sefton District of the West Derby Union. D. T. Key, M.R.C.S. and L.S.A.Lond., appointed Medical Officer and Public Vaccinator for the Wyke Regis District of the Weymouth Union. H. O'Farrell, L.R.C.P., L.R.C.S.Irel., appointed Medical Officer to the No. 1 Portumna Dispensary District. H. F. Stokes, M.D., appointed District Medical Officer, London County Council. H. J. Taylor, M.R.C.S., L.R.C.P.Lond., appointed Senior House Surgeon to the Manchester Royal Eye Hospital. F. Whitelaw, M.R.C.S., L.R.C.P.Lond., appointed Medical Officer for the Second District of the Holsworthy Union. F. S. Zaytoun, M.B., C.M.Edin., appointed Medical Officer for the Third District of the Bellingham Union.

**THE UNIFORM SYSTEM OF ACCOUNTS, AUDIT, AND TENDERS, for Hospitals and Institutions, with Certain Checks upon Expenditure; also the Index of Classification.** Compiled by a Committee of Hospital Secretaries, and adopted by a General Meeting of the same, 18th January, 1892. And certain Tender and other Forms for securing Economy, By Sir HENRY BURDETT, K.C.B. Demy 8vo, profusely illustrated with Specimen Tables, Index of Classification, Forms of Tender, &c., cloth extra, 6s.

**ACCOUNT BOOKS FOR INSTITUTIONS, designed in accordance with the Uniform System of Accounts for Hospitals and Institutions.** By Sir HENRY BURDETT, K.C.B. Being a complete set of Account Books ruled in accordance with the Uniform System adopted by the Metropolitan Hospital Sunday Fund.
(*Full Description of Books and Prices sent post free on application*)

London: THE SCIENTIFIC PRESS, Ltd., 28 & 29, Southampton Street, Strand, W.C.

### LATEST NURSING MANUALS.

Crown 8vo., profusely Illustrated, cloth gilt, 5s.

### A HANDBOOK FOR NURSES.

By J. K. WATSON, M.D., M.B., C.M.

"A concise and comprehensive exposition of as much medical knowledge as a nurse needs. The work cannot but prove useful for whom it has been designed."—*Scotsman.*

New Edition, profusely Illustrated, Crown 8vo., cloth gilt, 3s. 6d.

### NURSING: Its Theory and Practice.

Being a complete Text-book of Medical, Surgical, and Monthly Nursing.
By PERCY G. LEWIS, M.D., M.R.C.S., L.S.A.
With entirely new Chapters on Mental Nursing, Mechanical Therapeutics, Massage, Schott, Tallerman, and Weir-Mitchell's Treatments.

"We have read the book with interest, and can warmly recommend it as a nursing manual."—*Birmingham Medical Review.*

Crown 8vo., Illustrated, cloth gilt, 7s. 6d. net.

### PRACTICAL POINTS IN NURSING.

For Nurses in Private Practice. By EMILY A. M. STONEY.

"The hints given are excellent, and we hope they may be widely read."—*British Medical Journal.*

Crown 8vo., profusely Illustrated, cloth gilt, 6s.

### PHYSIOLOGY: Experimental and Descriptive.

By B. P. COLTON, Professor of Natural Science, Illinois University.

*Complete Catalogue of Works on Nursing and allied subjects sent post free on application.*

LONDON: THE SCIENTIFIC PRESS, LTD., 28 & 29, Southampton Street, Strand, W.C.

